# Educational Case: Hemolysis elevated liver enzymes and low platelets (HELLP syndrome)

**DOI:** 10.1016/j.acpath.2022.100055

**Published:** 2022-09-23

**Authors:** Larry Nichols, Kelsey Bree Harper, Keisha R. Callins

**Affiliations:** Mercer University School of Medicine, Macon, GA, USA

**Keywords:** Pathology competencies, Organ system pathology, Female reproductive system, Pregnancy, Preeclampsia, HELLP syndrome

## Abstract

Recommended management of patients with preeclampsia starts with a comprehensive clinical maternal and fetal evaluation, including maternal complete blood count, platelets, creatinine, LDH, liver enzymes, and urine test for proteinuria, along with fetal ultrasonographic evaluation and fetal antepartum testing.^7^ Subsequent management depends on the results of this evaluation and on gestational age. Continued observation is recommended for a woman with a preterm fetus if she has gestational hypertension or preeclampsia without severe features, until delivery at 37 weeks of gestation in the absence of abnormal antepartum testing, preterm labor, premature rupture of membranes, or vaginal bleeding.^7^ There are numerous conditions precluding such expectant management including severe hypertension refractory to treatment, persistent headaches refractory to treatment, epigastric or right upper pain refractory to treatment, visual disturbances, motor deficit, altered sensorium, stroke, myocardial infarction, new or worsening renal dysfunction, pulmonary edema, suspected acute placental abruption, vaginal bleeding in the absence of placenta previa, eclampsia, or HELLP syndrome.^7^

The following fictional case is intended as a learning tool within the Pathology Competencies for Medical Education (PCME), a set of national standards for teaching pathology. These are divided into three basic competencies: Disease Mechanisms and Processes, Organ System Pathology, and Diagnostic Medicine and Therapeutic Pathology. For additional information, and a full list of learning objectives for all three competencies, see https://www.journals.elsevier.com/academic-pathology/news/pathology-competencies-for-medical-education-pcme.^1^

## Primary objective

Objective FDP1.5: Eclampsia. Explain the principal pathophysiologic aberrations of the placenta and maternal circulation in preeclampsia and eclampsia; the characteristic morphologic features in the placenta, liver, kidney, and brain; and how management is affected by gestational age and severity of disease.

Competency 2: Organ System Pathology; Topic: Female Reproductive—Disorders of Pregnancy (FDP); Learning Goal 1: Disorders of Pregnancy.

## Secondary objective

Objective FDP1.3: Late pregnancy. Describe how disorders of late pregnancy can lead to effects that threaten the mother and/or fetus.

Competency 2: Organ System Pathology; Topic: Female Reproductive—Disorders of Pregnancy (FDP); Learning Goal 1: Disorders of Pregnancy.

## Patient presentation

A 41-year-old woman presents to a small rural hospital emergency department at 03:00, with the abrupt onset of severe acute epigastric pain. The pain is constant; it does not radiate outside of the mid-upper abdomen. The patient is pregnant, at 35 weeks gestation, with normal prenatal examination and blood pressure each visit through 34 weeks, and this is three days after the last prenatal examination. The patient has a past medical history of asthma, a remote appendectomy, a left oophorectomy for a cyst, a pre-term vaginal delivery at 34 weeks gestation 21 years ago, and no history of hypertension. Her asthma is quiescent, not requiring medication.

## Diagnostic findings, Part 1

On examination, the patient is found to have a blood pressure of 195/112 mm Hg. She is afebrile, without tachycardia or tachypnea. Her chest is clear to auscultation. Her abdomen has epigastric tenderness. She has no lower extremity edema.

## Questions/discussion points, Part 1

### What is the differential diagnosis for acute epigastric pain in late pregnancy?

Healthcare practitioners and medical students learning obstetrics may be tempted to assume that abdominal pain in a pregnant patient is due to the pregnancy, but it can be due to diseases unrelated to the pregnancy. Acute epigastric pain in late pregnancy can be due to peptic ulcer disease, gastritis, gastroesophageal reflux disease, cholecystitis, pancreatitis, hepatitis, acute fatty liver of pregnancy, or the hemolysis elevated liver enzymes and low platelets (HELLP) syndrome. Some features of epigastric pain may be suggestive of specific diagnoses. Colicky pain radiating to the right shoulder would be suggestive of cholecystitis related to gallstones. Pain radiating to the back would be suggestive of pancreatitis. Pain radiating to the right upper quadrant would be suggestive of hepatitis. It is important to remember that while some of the conditions in the differential diagnosis, such as peptic ulcer disease, are not specific to pregnancy, others, such as the HELLP syndrome, are specific to pregnancy. A focused history and physical examination will always be important in the initial evaluation.

### How can disorders of late pregnancy lead to effects that threaten the mother and/or fetus?

Acute fatty liver of pregnancy and HELLP syndrome are acute complications of late pregnancy that can be life-threatening to the mother and fetus.^2^ Acute fatty liver of pregnancy is a rare obstetric emergency with an uncertain exact cause, but emerging research suggests that it is sometimes due to autosomal recessive disorders of mitochondrial fatty acid beta-oxidation, particularly deficiencies in mitochondrial trifunctional protein and its alpha subunit long-chain 3-hydroxyacyl CoA dehydrogenase, which catalyzes the penultimate step in the mitochondrial beta-oxidation of long-chain fatty acids. Decreased maternal and placental fatty acid catabolism leads to an accumulation of 3-hydroxy fatty acid intermediates in the maternal circulation, resulting in hepatotoxicity and life-threatening liver failure.^2^

HELLP syndrome and preeclampsia seem to share early pathogenesis as a disorder of placenta formation later resulting in inadequate fetal perfusion and maternal vascular endothelial injury.^3^ HELLP syndrome probably represents a severe form of preeclampsia with particularly marked liver injury. In HELLP syndrome, as in preeclampsia, hepatic injury is precipitated by sinusoidal fibrin deposition and increased sinusoidal pressures leading to sinusoidal endothelial cell necrosis, further fibrin deposition, sinusoidal obstruction, and hepatocyte necrosis.^4^^,^^5^ This is associated with a systemic inflammatory response, a hypercoagulable state, microangiopathic hemolytic anemia, thrombocytopenia, and a risk of life-threatening hemorrhage, especially in the liver, which can then rupture.^6^ Premature separation of the placenta from the uterine lining, placental abruption, can lead to vaginal hemorrhage that threatens the mother and fetus, but this patient has no vaginal hemorrhage. Acute chorioamnionitis can lead to bacteremic infection that threatens the mother and fetus, but if it is threatening, this infection causes fever and foul-smelling vaginal discharge, which this patient does not report. Intrahepatic cholestasis of pregnancy carries a modest risk of fetal loss, but it presents with pruritis, followed in some cases by darkening urine, and occasionally light stools and jaundice, not the clinical history in this case.^6^

### How do the findings of physical examination alter the differential diagnosis?

The finding of severe hypertension quickly elevates preeclampsia to the top of the differential diagnosis. The new onset of hypertension after 20 weeks of gestation is essential to the diagnosis of preeclampsia.^3^ Hypertension is not a feature of acute fatty liver of pregnancy, while nausea, vomiting, polyuria, polydipsia, and encephalopathy, all absent in this case, are features of acute fatty liver of pregnancy.^2^ Preeclampsia is also approximately one thousand times more common than acute fatty liver of pregnancy.^2^^,^^3^ Hypertension is not a feature of peptic ulcer disease, gastritis, cholecystitis, pancreatitis, or hepatitis, so these diagnoses are all less likely with this finding.

### What laboratory tests might be helpful in narrowing the differential diagnosis?

Hemoglobin and hematocrit could help determine if the patient is bleeding from peptic ulcer disease or has hemolysis from the HELLP syndrome. A platelet count is essential in determining whether the patient has HELLP syndrome. A white blood cell count could help determine whether the patient has an inflammatory disorder; it is usually normal in patients with preeclampsia but often mildly elevated in those with HELLP syndrome. Liver enzyme levels are essential in determining whether the patient has HELLP syndrome. Glucose could help determine whether the patient has gestational diabetes mellitus. If blood glucose is not sufficiently elevated to diagnose gestational diabetes mellitus and the patient is not critically ill with severe hypertension, an oral glucose tolerance test could be done to determine if a patient has gestational diabetes mellitus. Blood urea nitrogen and creatinine could help determine if the patient has renal dysfunction from preeclampsia. Urinalysis could reveal proteinuria as evidence of renal dysfunction from preeclampsia.

## Diagnostic findings, Part 2

The patient's initial blood test results are shown in Table 1. Urinalysis shows 3+ proteinuria. The patient is treated with labetalol and magnesium sulfate and airlifted to a large urban hospital.

**Table 1:** Initial blood test results.

	Patient	Reference range
White blood cell count	12,000/mm^3^	4000–10,000/mm^3^
Platelet count	157,000/mm^3^	140,000–440,000/mm^3^
Hemoglobin	13.4 g/dL	11.7–15.7 g/dL
Hematocrit	38.7%	36–46%
Creatinine	1.0 mg/dL	0.8–1.5 mg/dL
Glucose	104 mg/dL	70–110 mg/dL
Bilirubin	0.6 mg/dL	0.3–1.5 mg/dL
Alanine aminotransferase (ALT)	139 U/L	<40 U/L
Aspartate aminotransferase (AST)	133 U/L	<40 U/L
Lactate dehydrogenase (LDH)	296 U/L	<170 U/L

## Questions/discussion points, Part 2

### What is the interpretation of the initial blood test results as shown in Table 1?

The white blood cell count is mildly elevated, which can be a feature of HELLP syndrome, but is non-specific. A mildly elevated white blood cell count could also be due to peptic ulcer disease, gastritis, cholecystitis, pancreatitis, hepatitis, or acute fatty liver of pregnancy. The platelet count is normal, which excludes the diagnosis of HELLP syndrome. The hemoglobin and hematocrit are normal, which generally suggests a patient has not had significant bleeding, but this does not exclude the possibility of hemorrhage in this case because there may not have been enough time for compensatory hemodilution (replacement of lost red blood cells by water from extravascular compartments). The creatinine is normal, but this does not exclude the possibility of acute kidney injury because it is a lagging indicator. Creatinine typically takes a day or so to rise following acute kidney injury. The glucose is normal, so gestational diabetes mellitus is unlikely. Hypoglycemia can be a feature of acute fatty liver of pregnancy, so a normal serum glucose makes this less likely. The liver enzymes are elevated, which could be from hepatitis, but these enzymes are also present in kidney, heart, skeletal muscle, pancreas, lung, and many other organs, which can release these enzymes when they are injured. Lactate dehydrogenase (LDH) is more elevated than the other enzymes in these results, which is suggestive of hemolysis because blood cells have an abundance of this enzyme, but the bilirubin is normal. Hemoglobin breakdown from hemolysis results in excess bilirubin, eventually overwhelming the liver's ability to excrete it, so that serum bilirubin rises. The elevated serum LDH without elevated bilirubin could represent early hemolysis. This indicates a need for close follow-up testing.

### Does this patient have preeclampsia, eclampsia, or HELLP syndrome?

The maternal vascular endothelial injury of preeclampsia is generally first manifested by hypertension. The next most common manifestation is proteinuria, which is correlated with glomerular endotheliosis characterized by enlarged bloodless glomeruli with capillary lumens occluded by swollen endothelial and sometimes mesangial cells.^3^ Other manifestations include renal insufficiency, pulmonary edema, new-onset headache, elevated liver enzyme levels, and thrombocytopenia. Elevated liver enzyme levels and thrombocytopenia can be features of preeclampsia, with or without HELLP syndrome. The American College of Obstetricians and Gynecologists criteria for preeclampsia, simplified here, are new-onset hypertension after 20 weeks of gestation and any of the following: proteinuria, thrombocytopenia, impaired liver function, renal insufficiency, pulmonary edema, or new-onset headache.^7^^,^^8^ This patient has new-onset hypertension after 20 weeks of gestation and proteinuria, which fulfills these criteria for preeclampsia. Eclampsia is defined by new-onset seizures in a patient with pregnancy and hypertension in the absence of other seizure-causing conditions, such as epilepsy, cerebral arterial ischemia and infarction, intracranial hemorrhage, or drug use. A significant proportion of women (20–38%) do not demonstrate the classic signs of preeclampsia (hypertension or proteinuria) before the seizure episode.^7^ This patient does not have seizures and hence does not have eclampsia.

The maternal vascular endothelial injury of the HELLP syndrome leads to the formation of fibrin strands crossing capillary lumens, which trap platelets and shear off fragments of red blood cells when they are pushed through the capillaries. This causes thrombocytopenia and microangiopathic hemolysis.^3^ Hemolysis releases LDH from damaged erythrocytes. Liver injury releases aspartate aminotransferase (AST) from damaged hepatocytes. The components of the HELLP syndrome are in the name of the condition, but there are multiple classification systems with specific diagnostic criteria. The widely used Tennessee criteria for complete HELLP syndrome, simplified here, are LDH >600 U/L, AST >70 U/L, and platelets <100,000/mm^3^.^4^^,^^7^ This patient does not fulfill the criteria for HELLP syndrome.

It is important for healthcare practitioners and students acquiring competency in obstetrics to appreciate that preeclampsia can be severe and rapidly progress to a life-threatening condition without fulfilling criteria for eclampsia or HELLP syndrome. In a case like this one, it is more urgent to recognize that the patient is severely ill and arrange higher level care if needed than to contemplate what tests to order for various specific diagnoses.

## Diagnostic findings, Part 3

On admission to the large urban referral hospital, the patient complains of a headache and continued abdominal pain. Her temperature is 37.4 °C, pulse rate 74 beats/min, blood pressure 148/80 mm Hg, and respiratory rate 20 breaths/min. On examination, the patient has epigastric tenderness, bloody fluid on the perineum, ruptured placental membranes, and no edema. The blood test results at 10:30, 7.5 h after initial presentation, are shown in Table 2.

**Table 2:** Blood test results 7.5 h after initial presentation.

	Patient	Reference range
Platelet count	30,000/mm^3^	140,000–440,000/mm^3^
Hemoglobin	11.5 g/dL	11.7–15.7 g/dL
Hematocrit	32.5%	36–46%
White blood cell count	16,800/mm^3^	4000–10,000/mm^3^
Prothrombin time (PT)	19.7 seconds	10.5–13 seconds
Partial thromboplastin time (PTT)	34.9 seconds	25–33 seconds
Bilirubin	4.3 mg/dL	0.3–1.5 mg/dL
Conjugated bilirubin	1.8 mg/dL	0.0–0.4 mg/dL
Blood urea nitrogen (BUN)	15 mg/dL	9–20 mg/dL
Creatinine	1.0 mg/dL	0.8–1.5 mg/dL
Fibrinogen	140 mg/dL	233–496 mg/dL

## Questions/discussion points, Part 3

### What is the interpretation of the follow-up blood test results as shown in Table 2?

The platelet count has fallen dramatically in the 7.5 h since the patient presented. This could be from bleeding or autoimmune platelet destruction, but in the context of this case, the far more likely etiology is the consumption of platelets in clotting within liver sinusoids and small blood vessels elsewhere in the body, which is characteristic of the HELLP syndrome. The hemoglobin and hematocrit have slipped below the lower limits of normal but have not fallen very much compared to the platelet count. This could be from bleeding or hemodilution (due intravenous fluid administration), but in the context of this case, the more likely etiology is hemolysis as part of the HELLP syndrome. The white blood cell count has risen from mildly elevated to what might be regarded as moderately elevated, which is most likely part of the stress response to severe illness mediated by epinephrine and cortisol. The prothrombin time and partial thromboplastin time are prolonged, which is most likely due to the consumption of protein blood clotting factors in coagulation within liver sinusoids and small blood vessels elsewhere in the body. Elevated prothrombin time and partial thromboplastin time are not part of the HELLP syndrome; when they in occur in a patient with HELLP syndrome, this is most likely from disseminated intravascular coagulation (DIC) in addition to the HELLP syndrome. The bilirubin has risen dramatically, most likely due to hemolysis, but also partly from injured hepatocytes releasing their contents into the bloodstream. The blood urea nitrogen and creatinine are normal, which does not exclude the possibility of acute kidney injury because these values take time to rise. The fibrinogen is low, most likely from consumption in clotting, which most likely represents progression to DIC in addition to the HELLP syndrome.

### What is the diagnosis and most appropriate management of this patient on admission to the large urban referral hospital?

The patient has developed thrombocytopenia, coagulopathy, and hyperbilirubinemia. This could be classified as severe preeclampsia.^7^^,^^9^ Features that make preeclampsia severe, simplified here, includ systolic blood pressure of 160 mm Hg or more, diastolic blood pressure of 110 mm Hg or more, thrombocytopenia (platelet count of less than 100,000/mm^3^), liver enzymes more than twice the upper limit of normal, severe persistent right upper quadrant or epigastric pain, renal insufficiency, pulmonary edema, refractory headache, or visual disturbances.^7^ The most appropriate management is to deliver the baby.^3^^,^^7^ Delivering the baby and the placenta removes the anatomic source of the condition. The risks and complications of delivery are outweighed by the risks and complications of severe preeclampsia. One might ask why not deliver the baby at the hospital where the patient first presented. A substantial and increasing proportion of small rural hospitals in the USA have no obstetric service, and those that do often have limited blood transfusion and neonatal resources. Even if a small rural hospital has an obstetric service, it might take nearly as long for an obstetrician to reach that hospital as it would take to airlift the patient to a referral hospital.

## Diagnostic findings, Part 4

**Fig. 1:**
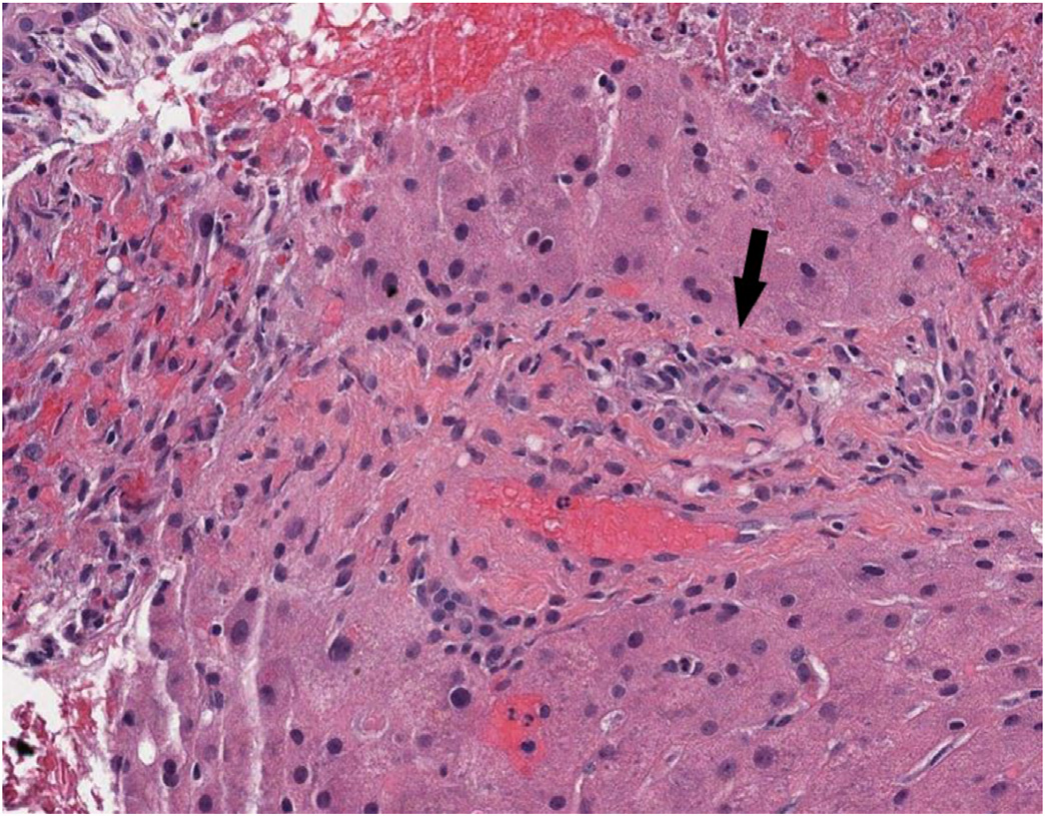
HELLP syndrome liver findings. The portal tract in the center (indicated by the arrow), roughly outlined by wavy pink collagen and containing a congested vein, shows mild infiltration by lymphocytes and neutrophils, with hepatocyte necrosis, hemorrhage, and neutrophilic response in the periportal parenchyma on the left and upper right. H&E, intermediate power.

**Fig. 2:**
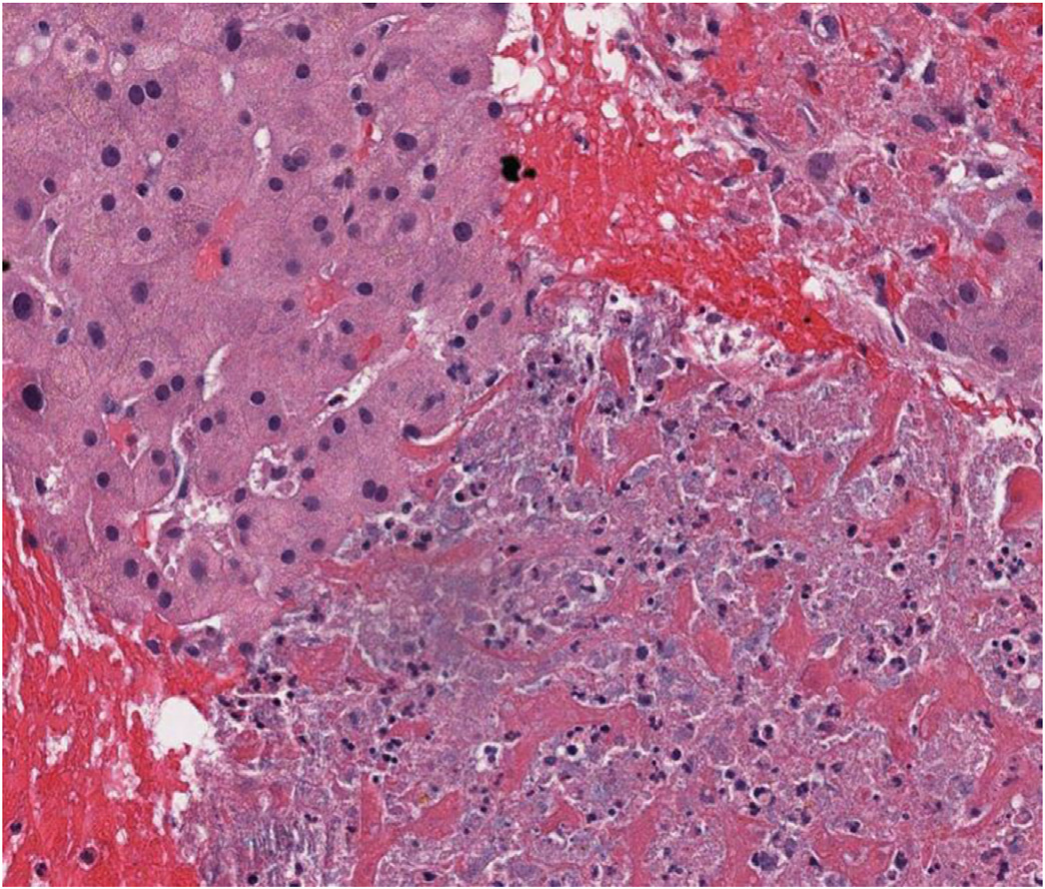
HELLP syndrome liver findings. The lobular parenchyma in the lower right shows sinusoidal fibrin deposits, coagulative necrosis, basophilic debris, neutrophilic response, and adjacent areas of hemorrhage. H&E, intermediate power.

**Fig. 3:**
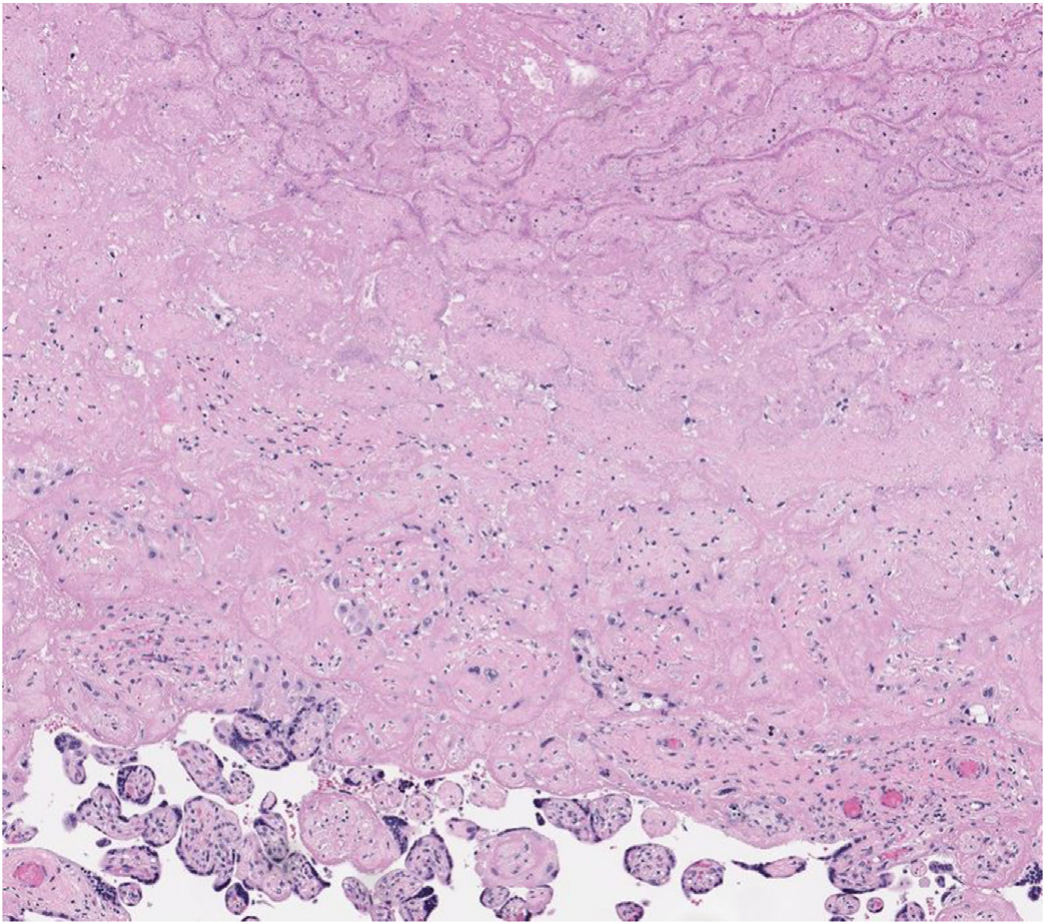
HELLP syndrome placental findings. A villous infarct, with sharply circumscribed edge, collapse of the intervillous spaces, intervillous fibrin accumulation, loss of villous blood vessels, and presence of karyorrhectic debris, occupies the upper and middle portions of the image. Adjacent villi have increased syncytial knots visible as dark blue-black rims. H&E, intermediate power.

**Fig. 4:**
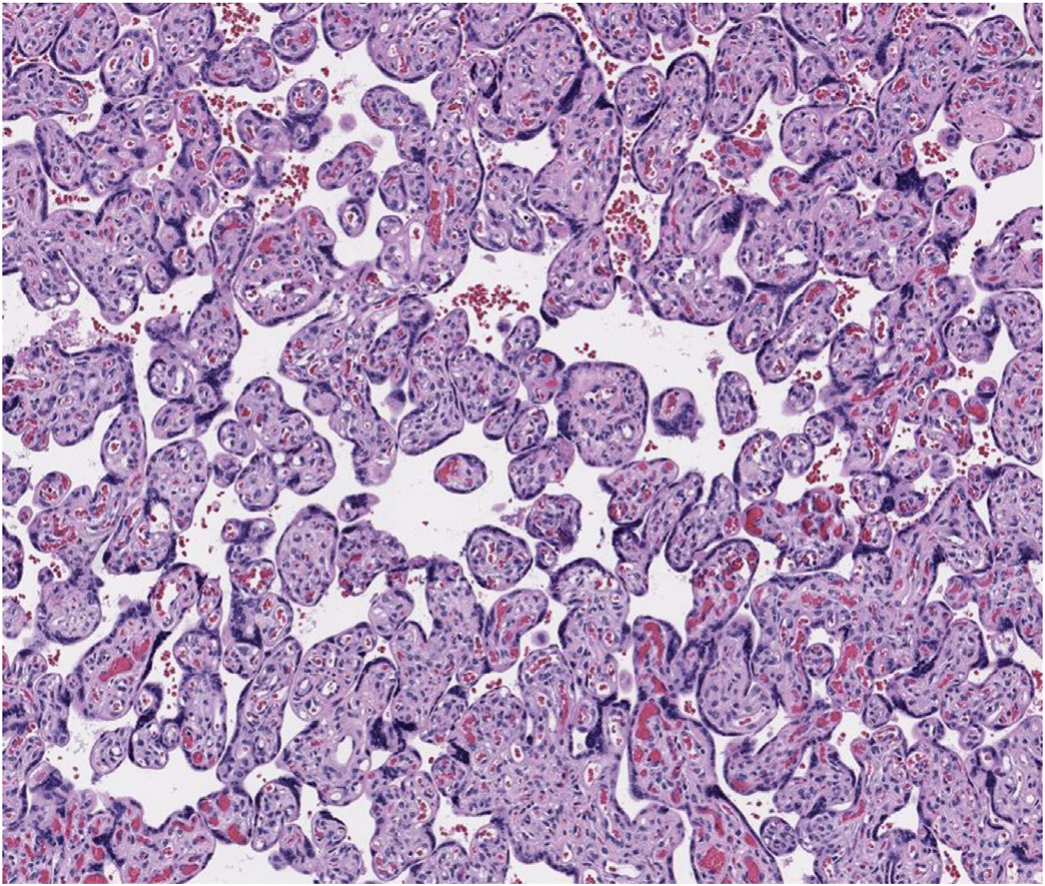
Normal placenta. Mature villi, with a moderate amount of non-edematous stroma, blood vessels containing blood, and moderate numbers of syncytial knots, separated by intervillous spaces with blood and no fibrin accumulation and are evident in third-trimester placenta. H&E, intermediate power.

An emergency cesarean section is performed after the patient is transfused platelets and fresh frozen plasma to prevent excessive bleeding during surgery. The baby is delivered, with Apgar scores of 8 at 1 min and 10 at 5 min, and then transferred to the neonatal intensive care unit in stable condition. The mother's blood test results following delivery are shown in Table 3. The markedly elevated LDH and AST, together with the low platelet count, fulfill criteria for a diagnosis of HELLP syndrome. Liver biopsy is not needed for the diagnosis of HELLP syndrome, but if an intraoperative liver biopsy would have been done at the time of cesarean section in this case, it could have shown the findings in Figs. 1 and 2. The placenta at the time of delivery is sent to pathology for examination. The placenta is small for gestational age. The microscopic findings are shown in Fig. 3. Normal placenta at the same gestational age is shown in Fig. 4 for comparison.

**Table 3:** Blood test results following delivery.

	Patient	Reference range
White blood cell count	5000/mm^3^	4000–10,000/mm^3^
Platelet count	69,000/mm^3^	140,000–440,000/mm^3^
Hemoglobin	6.9 g/dL	11.7–15.7 g/dL
Hematocrit	19.2%	36–46%
Prothrombin time (PT)	18.3 seconds	10.5–13 seconds
Partial thromboplastin time (PTT)	38.4 seconds	25–33 seconds
Bilirubin	5.3 mg/dL	0.3–1.5 mg/dL
Alkaline phosphatase	133 U/L	40–125 U/L
Alanine aminotransferase (ALT)	1882 U/L	<40 U/L
Aspartate aminotransferase (AST)	3306 U/L	<40 U/L
Lactate dehydrogenase (LDH)	11,908 U/L	<170 U/L

## Questions/discussion points, Part 4

Please interpret the blood test results following delivery as shown in Table 3.

The white blood cell count has decreased and is now low normal. This is most likely because the stress response to severe illness has abated, but patients with overwhelming sepsis can have normal white blood cell counts falling from elevated levels before the sepsis became overwhelming. The platelet count is improved from the preoperative pretransfusion count, but the production of sufficient replacement platelets by the patient's bone marrow megakaryocytes will likely take at least a few days. The hemoglobin and hematocrit are substantially lower than on admission, presumably due to hemolysis, but it is reasonable to hope that these represent a nadir from which they will gradually recover. The prothrombin time and partial thromboplastin time are still prolonged, but it is a reasonable hope that the liver will recover and gradually replace the clotting factors consumed in coagulation. Bilirubin remains elevated due to the combined effect of hemolysis generating excess and bilirubin and liver injury releasing it from damaged hepatocytes. The marked elevation of ALT, AST, and LDH, with LDH much higher than the AST and ALT, can be attributed to the HELLP syndrome.

### Describe the findings in the placenta

The placenta demonstrates villous infarcts, as shown in Fig. 3, and accelerated maturation manifested by increased syncytial knots, microscopic findings of maternal vascular malperfusion, which can be associated with preeclampsia and HELLP syndrome.^10^^,^^11^

### What is the role of pathologic examination of the placenta in HELLP syndrome?

Pathologic examination of the placenta often helps to explain adverse outcomes, such as fetal growth restriction, preterm birth, fetal brain injury, or intrauterine fetal demise.^10^ Pathologic examination of the placenta can help support or confirm a diagnosis of HELLP syndrome, or it can reveal an alternative or additional diagnosis, such as toxoplasmosis, syphilis, or cytomegalovirus infection. The most common pathologic findings associated with HELLP syndrome are those of maternal vascular malperfusion, which include accelerated villous maturation, distal villous hypoplasia, crowded villi with large dense syncytial knots, agglutinated in intervillous fibrin, villous infarction, and hematoma.^10^ Such findings are nearly as common in severe preeclampsia without HELLP syndrome as in HELLP syndrome.^11^ These nonspecific findings of placental injury are also the most common findings in gestational diabetes mellitus.^12^ There are no pathologic findings specific for HELLP syndrome or preeclampsia. Perhaps, the most important role of pathologist examination of the placenta in HELLP syndrome is to rule out infection as an alternative or additional diagnosis.

Finding neutrophils in the membranes (acute chorioamnionitis) or villi (acute villitis) would be indicative of a maternal inflammatory response to acute infection.^11^ If neutrophils are observed in the umbilical cord vessels in addition to the chorionic plate and amniotic membranes, this would represent a fetal inflammatory response as well and more severe infection. Acute villitis is uncommon and is associated with listeriosis. Finding lymphocytes in villi would be suggestive of villitis of unknown etiology, which most often represent a maternal T-cell-mediated immune response to antigens in the fetal villus stroma, but can be a feature of transplacental infection with TORCH agents (toxoplasmosis, other [syphilis], rubella, cytomegalovirus, and herpes simplex virus).^11^ Finding plasma cells among the lymphocytes would favor the diagnosis of transplacental infection.^11^

### Describe the findings in the liver biopsy

The liver biopsy demonstrates sinusoidal fibrin deposition, coagulative necrosis of hepatocytes, basophilic debris, neutrophilic response, and adjacent areas of hemorrhage within the lobular parenchyma, along with mild portal infiltration by lymphocytes and neutrophils, which are the microscopic liver pathology findings of HELLP syndrome. Such findings can be seen in severe preeclampsia, but the lack of steatosis makes acute fatty liver of pregnancy highly unlikely.

### What pathophysiologic sequence led to this patient's syndrome?

This patient's syndrome is preeclampsia that rapidly evolves into HELLP syndrome. Placental dysfunction is key in the pathophysiology of both preeclampsia and HELLP syndrome.^3^ Placental development starts with extravillous trophoblast cell proliferation, migration, and invasion into the decidua underneath the implantation site, orchestrated by transcription factors, released by extravillous trophoblastic cells, decidualized stromal cells, and uterine natural killer cells, which remodels spiral arteries to grow their capacity. In patients with preeclampsia, with or without HELLP syndrome, impaired extravillous trophoblast cell development and defective spiral artery remodeling result in a placenta with insufficient functional capacity. This dysfunctional placenta releases anti-angiogenetic factors, necrotic debris, and cell-free DNA that induces systemic endothelial dysfunction. One anti-angiogenic factor in preeclampsia is soluble fms-like tyrosine kinase 1 (sFLT).^3^ In a normal healthy pregnancy, vascular homeostasis is maintained by physiological levels of vascular endothelial growth factor (VEGF) and placental growth factor (PlGF) signaling by binding to fms-like tyrosine kinase 1 (FLT1) receptors expressed in endothelial cell membranes. In preeclampsia, excess soluble FLT1 (sFLT1), a truncated version lacking the portion that anchors in the endothelial cell membrane, is released by the placenta. Circulating sFLT1 binds VEGF and PlGF in the circulation, inhibiting VEGF and PlGF signaling via endothelial cells in the vasculature. This inhibition results in endothelial cell dysfunction, with reduced production of prostacyclin and nitric oxide, and the release of procoagulant proteins.^3^ Preeclampsia and HELLP syndrome are also associated with a systemic inflammatory response, which contributes to the pro-thrombotic condition, with microthrombi formation and fibrin deposition. In the liver in HELLP syndrome, fibrin deposition eventually results in the obstruction of the hepatic sinusoids, ischemia, and progressive hepatocyte necrosis.^5^

### What are salient epidemiological features of preeclampsia and HELLP syndrome?

Preeclampsia occurs in 4%–5% of all pregnancies.^3^ The incidence of preeclampsia has been increasing; it rose 25% between 1987 and 2003 in the USA.^7^ Risk factors for preeclampsia include nulliparity, multifetal pregnancy, advanced maternal age, in vitro fertilization, previous preeclampsia, and maternal comorbidities (obesity, diabetes mellitus, chronic hypertension, kidney disease, and systemic lupus erythematosus).^7^ Preeclampsia heritability is estimated to be approximately 55%. There is compelling evidence that alterations near the FLT1 locus in the human fetal genome may be causal in the development of preeclampsia.^13^ Approximately 90% of cases present at 34 weeks gestation or later, including up to 5% presenting postpartum.^14^ In the USA, the incidence of hypertensive disorders of pregnancy has risen, with African-American women at higher risk of associated mortality than Hispanic, American-Indian, White, and Asian or Pacific-Islander women.^3^

Eclampsia is defined by new-onset seizures in the absence of other causative conditions.^7^ Intrapartum magnesium sulfate treatment reduces the risk of new-onset seizures in patients with severe preeclampsia.^7^ The incidence of eclampsia is very low (1.9% of patients with preeclampsia) and decreasing in the setting of more widespread antenatal care and use of magnesium sulfate.^3^^,^^7^

HELLP syndrome occurs in 0.5%–0.9% of all pregnancies and in 10%–20% of those with severe preeclampsia.^15^ In about 70% of cases, HELLP syndrome develops before delivery, but it can occur within 48 h postpartum. The average age of women with HELLP syndrome is higher than in those with preeclampsia or uncomplicated pregnancy.^15^ This patient presented at 35 weeks gestation; signs and symptoms of HELLP syndrome typically develop between 27 and 37 weeks of gestation.^15^ Most cases occur in the third trimester, but it can also occur or worsen postpartum. It is unknown how and why some cases of HELLP syndrome and preeclampsia occur in the first few days postpartum. It is speculated that perhaps the process of delivery may release anti-angiogenetic factors, necrotic debris, and cell-free DNA sequestered in the placenta until delivery, and then these substances induce maternal systemic endothelial dysfunction.^3^

### What are the clinical manifestations of preeclampsia and the HELLP syndrome?

The new onset of hypertension after 20 weeks of gestation is the single clinical manifestation essential to the diagnosis of preeclampsia.^7^^,^^8^ For this diagnosis, hypertension is defined as a systolic blood pressure of 140 mm Hg or more or a diastolic blood pressure of 90 mm Hg or more, or both, on two occasions at least 4 h apart after 20 weeks of gestation in a woman with a previously normal blood pressure.^7^ Women with severe gestational hypertension (systolic 160 mm Hg or higher, or diastolic 110 mm Hg or higher) should be diagnosed with preeclampsia with severe features. Traditionally, the new onset of proteinuria was also regarded as essential to the diagnosis of preeclampsia, but this is not always present when other clinical manifestations of maternal vascular endothelial injury are, so the criteria have been broadened to include any one of multiple other manifestations: thrombocytopenia, impaired liver function, renal insufficiency, pulmonary edema, or new-onset headache.^7^^,^^8^

Rapid onset of severe persistent epigastric or right upper quadrant abdominal pain is the most common symptom of HELLP syndrome.^15^, ^16^, ^17^ This abdominal pain is thought to be from hepatic inflammation and edema stretching the liver capsule, where pain nerve endings reside.^7^ The most common symptoms of HELLP syndrome, after abdominal pain, include nausea, vomiting, and malaise, but other symptoms include headache, along with visual disturbances, excessive weight gain, and generalized edema. Notice that this patient presented at 03:00. The symptoms of HELLP syndrome are characteristically worse at night and recede during the day. This patient presented with the new onset of hypertension; hypertension is the most common sign of HELLP syndrome.^18^ This patient presented with proteinuria, which is the most common laboratory abnormality (excluding those that define the condition).^16^^,^^18^ Hypertension and proteinuria are cardinal features of preeclampsia, and the great majority of patients with HELLP syndrome have preeclampsia.^15^^,^^17^^,^^18^ Although most patients have hypertension and proteinuria, neither is required for the diagnosis of HELLP syndrome. The evolution of HELLP syndrome from preeclampsia is often sudden.

### What is the differential diagnosis for preeclampsia and HELLP syndrome?

The differential diagnosis for preeclampsia is broad and incudes methamphetamine toxicity, alcohol withdrawal, acute kidney disease, thrombotic microangiopathy, pheochromocytoma, and systemic lupus erythematosus.^19^^,^^20^ HELLP syndrome also has a broad differential diagnosis.^15^ Some of the differential diagnoses of HELLP syndrome include viral hepatitis, cholangitis, upper urinary tract infection, acute gastritis, acute pancreatitis, thrombotic thrombocytopenic purpura (TTP), hemolytic uremic syndrome, systemic lupus erythematosus, and antiphospholipid syndrome. It is important to exclude these alternate diagnoses, since none of them are managed like HELLP syndrome.

Fever would have brought TTP, pyelonephritis, and lupus into the differential diagnosis in this case, but this patient did not have fever. Fever is a feature of TTP; other features include hemolysis, thrombocytopenia, renal insufficiency, and altered mental status.^15^ TTP is due to deficiency of the von Willebrand factor-cleaving protease ADAMTS13 (a disintegrin and metalloproteinase with a thrombospondin type 1 motif member 13). Low levels of ADAMTS13 activity in adults are most often due to antibodies, which can be removed by plasma exchange.^15^ Fever is a feature of upper urinary tract infection (pyelonephritis); other features include flank pain, dysuria, malaise, nausea, and vomiting. The treatment of pyelonephritis is intravenous antibiotics. Fever can be a feature of systemic lupus erythematosus, which is primarily a disease of women of child-bearing age. There are dozens of other features of lupus, including hemolytic anemia, thrombocytopenia, leukopenia, proteinuria, renal insufficiency, and skin rashes.^15^ Lupus can be diagnosed by various tests for autoantibodies and can be treated with immunosuppressive therapy.

Acute fatty liver of pregnancy is also in the differential diagnosis for HELLP syndrome. Patients with HELLP syndrome usually have proteinuria and patients with acute fatty liver of pregnancy usually do not, so this can be a differentiating feature.^15^ Leukocytosis, hypoglycemia, elevated serum uric acid, and elevated ammonia are features of acute fatty liver of pregnancy and not typical features of HELLP syndrome, so these laboratory test abnormalities can help make this differentiation.^2^ Treatment, however, is the same: delivery of the baby.

### What is the recommended management for preeclampsia and HELLP syndrome?

Recommended management of patients with HELLP syndrome requires the availability of neonatal and obstetric intensive care units and personnel with special expertise, so patients with HELLP syndrome who are remote from term should receive care at a tertiary care center.^7^ The laboratory test components of the syndrome (LDH, transaminases, and platelet count) should be monitored frequently, at least every 12 h. Immediate delivery regardless of gestational age is often recommended.^7^

### What are the maternal and fetal outcomes of preeclampsia and HELLP syndrome?

Women with preeclampsia are at risk for developing acute kidney injury, placental abruption, pulmonary edema, cerebral hemorrhage, hepatic failure or rupture, stroke, cardiac failure, or progression to eclampsia.^8^ Approximately 6–7% of patients with preeclampsia develop acute kidney injury, 1–1.5% placental abruption, and 0.4–0.5% pulmonary edema; the other complications are less common.^8^ The risk of maternal death is low, estimated to currently be 6.6 per 1 million live births in the USA, decreased by approximately 50% over the past 20 years.^21^ Although the risk of maternal death is low, 10–15% of those from obstetric complications of pregnancy are associated with preeclampsia/eclampsia. Growing evidence suggests that women with preeclampsia have a long-term higher risk of stroke, ischemic heart disease, and chronic kidney disease, but this association may not represent long-term sequelae of preeclampsia. Instead, preeclampsia may be linked to these later diseases as an effect, not a cause, of underlying vascular disease that causes them all.^3^

The risk of adverse outcomes for the fetus is primarily from preterm delivery. Preeclampsia is associated with a risk of neonatal respiratory distress syndrome and bronchopulmonary dysplasia.^3^ Preeclampsia is associated with a reduced risk of the retinopathy of prematurity, perhaps related to the anti-angiogenic environment during preeclampsia.^3^

Women with HELLP syndrome are at risk for developing pulmonary edema, acute respiratory distress syndrome, and renal failure. In a study of 34 cases from 2005 through 2014, three mothers (8.8%) developed hepatic and renal failure, and one (2.9%) suffered intracerebral hemorrhage, but pulmonary complications were not reported; there were no maternal deaths, but perinatal death occurred in five cases (14.7%).^17^ In a study of 77 cases from 2008 to 2015, 35 of the neonates (45.5%) had adverse outcomes, including 17 (22.1%) with respiratory morbidity (respiratory distress syndrome and/or ventilatory support), 12 (15.6%) with low birthweight <5th percentile, 5 (6.5%) with sepsis, 5 (6.5%) requiring blood transfusion, 2 (2.6%) with cerebral morbidity (hemorrhage and/or seizures), and 1 with necrotizing enterocolitis; there were no neonatal deaths.^10^ In a third study of 43 patients from 2009 through 2016, 3 mothers (7%) had DIC, 2 impaired renal function, and 1 pleural effusion; there were 2 stillbirths, but no neonatal or maternal deaths.^16^ The current maternal mortality rate from HELLP syndrome is probably less than one percent.^16^^,^^17^ It is important to counsel the mother that after a pregnancy with HELLP syndrome, the risk of all forms of preeclampsia is increased in subsequent pregnancies.^16^

## Teaching points

• Preeclampsia is a common complication of pregnancy defined by hypertension with evidence of end-organ dysfunction.
• Evidence of end-organ dysfunction from preeclampsia includes proteinuria, renal insufficiency, new-onset headache, thrombocytopenia, impaired liver function, or abdominal pain.
• Eclampsia is a rare complication of pregnancy defined by hypertension and the new onset of seizures in the absence of other underlying seizure-causing conditions.
• In preeclampsia, excess soluble fms-like tyrosine kinase 1 (sFLT1), a truncated version of the endothelial cell receptor for vascular endothelial growth factor (VEGF), and placental growth factor (PlGF), lacking the portion that anchors in the endothelial cell membrane, is released by the placenta, binds circulating VEGF and PlGF, and inhibits VEGF and PlGF signaling that is needed for placental vascular homeostasis.
• In preeclampsia, failure of placental vascular homeostasis leads to a pro-thrombotic condition, with microthrombi formation and fibrin deposition, associated with a systemic inflammatory response.
• Acute abdominal pain in late pregnancy can be from a complication of pregnancy but may be due to a disease unrelated to pregnancy.
• The hemolysis elevated liver enzymes and low platelets (HELLP) syndrome is a complication of late pregnancy that can threaten the lives of mother and fetus.
• The most common symptom in HELLP syndrome is rapid onset of severe persistent abdominal pain in the epigastrium or right upper quadrant.
• Malaise, nausea, and vomiting are also common symptoms of HELLP syndrome.
• HELLP syndrome evolves, often suddenly, from preeclampsia, with hypertension and proteinuria, in the majority of patients.
• The laboratory test features of the HELLP syndrome are contained in the name of the condition.
• The placental findings of preeclampsia are those of maternal vascular malperfusion and are not specific for preeclampsia.
• In HELLP syndrome in the liver, fibrin deposition eventually results in obstruction of the hepatic sinusoids, ischemia, and progressive hepatocyte necrosis.
• Laboratory testing to differentiate HELLP syndrome from severe preeclampsia is often less important than recognizing the patient has one of these syndromes because they are both obstetric emergencies with the same treatment.
• Immediate delivery regardless of gestational age is often recommended for patients with HELLP syndrome.

## Funding

The article processing fee for this article was funded by an Open Access Award given by the Society of ‘67, which supports the mission of the Association of Pathology Chairs to produce the next generation of outstanding investigators and educational scholars in the field of pathology. This award helps to promote the publication of high-quality original scholarship in *Academic Pathology* by authors at an early stage of academic development.

## Declaration of competing interest

The authors declare that they have no known competing financial interests or personal relationships that could have appeared to influence the work reported in this paper.
